# Identifying threshold responses of Australian dryland rivers to future hydroclimatic change

**DOI:** 10.1038/s41598-020-63622-3

**Published:** 2020-04-20

**Authors:** Z. T. Larkin, T. J. Ralph, S. Tooth, K. A. Fryirs, A. J. R. Carthey

**Affiliations:** 1 Department of Earth and Environmental Sciences, Macquarie University, North Ryde, 2109 NSW Australia; 2 Department of Geography and Earth Sciences, Aberystwyth University, Aberystwyth, SY23 3DB UK; 3 Department of Biological Sciences, Macquarie University, North Ryde, 2109 NSW Australia

**Keywords:** Geomorphology, Hydrology, Climate-change impacts

## Abstract

Rivers provide crucial ecosystem services in water-stressed drylands. Australian dryland rivers are geomorphologically diverse, ranging from through-going, single channels to discontinuous, multi-channelled systems, yet we have limited understanding of their sensitivity to future hydroclimatic changes. Here, we characterise for the first time the geomorphology of 29 dryland rivers with catchments across a humid to arid gradient covering >1,800,000 km^2^ of continental eastern and central Australia. Statistical separation of five specific dominantly alluvial river types and quantification of their present-day catchment hydroclimates enables identification of potential thresholds of change. Projected aridity increases across eastern Australia by 2070 (RCP4.5) will result in ~80% of the dryland rivers crossing a threshold from one type to another, manifesting in major geomorphological changes. Dramatic cases will see currently through-going rivers (e.g. Murrumbidgee, Macintyre) experience step changes towards greater discontinuity, characterised by pronounced downstream declines in channel size and local termination. Expanding our approach to include other river styles (e.g. mixed bedrock-alluvial) would allow similar analyses of dryland rivers globally where hydroclimate is an important driver of change. Early identification of dryland river responses to future hydroclimatic change has far-reaching implications for the ~2 billion people that live in drylands and rely on riverine ecosystem services.

## Introduction

Rivers are lifelines in climatically variable and water-stressed drylands, the dry subhumid through hyperarid environments that cover 40–50% of the Earth’s land surface and host ~28% of the world’s population^1,2^. Dryland rivers are fundamentally important for human populations, providing a plethora of provisioning, regulating, supporting and cultural ecosystem services^1,3^. Yet dryland rivers exist in marginal environments and are threatened by declines in water availability due to the impacts of climate change (e.g. decreased rainfall, increased temperature and evapotranspiration, and greater climatic variability) and other human activities (e.g. river regulation, flow diversion and abstraction, and land use change)^4–6^. Rivers are not static conduits of water, sediment and nutrients, but adjust dynamically to a suite of internal and external drivers. Among various external drivers (e.g. tectonic activity, sea level fluctuations, climate), research has shown that late Quaternary hydroclimatic changes have driven substantial geomorphological changes to many dryland rivers globally, including during the mid to late Holocene [e.g.^7–10^]. Indeed, in tectonically stable settings such as continental Australia, and in reaches where rivers are free from significant bedrock influence, hydroclimatic changes are the principal driver of river response and resulting channel-floodplain geomorphology. To date, however, assessment of the potential likelihood and pathways of hydrological and geomorphological changes in dryland rivers due to future hydroclimate change have not been considered in any rigorous or systematic fashion^11,12^. Analyses of future changes have tended to focus solely on dryland hydrology, such as surface water availability or river flow regimes [e.g.^5,13–15^], rather than on the implications of these changes for river response and physical structure (e.g. number of channels, sinuosity, lateral stability, landform assemblages). This is a critical knowledge gap, as dryland river geomorphology provides the physical template atop which complex ecosystems and anthropogenic land uses operate and intersect^16^, thereby defining the range and quality of ecosystem service delivery.

**Figure 1:**
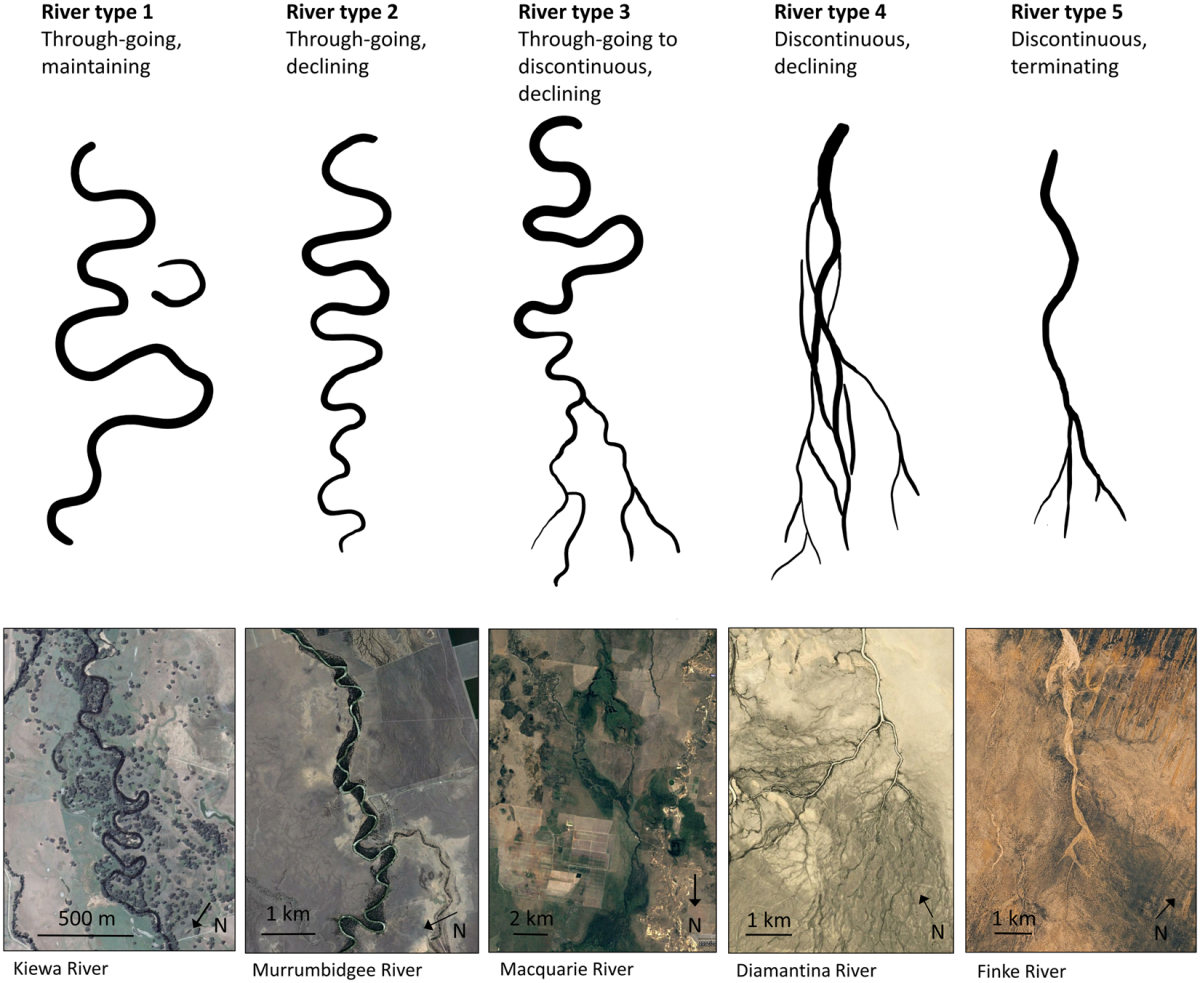
Schematic of the five dryland river types defined in this study, with satellite images of typical examples from various Australian rivers (Satellite imagery data: Google Earth, Image © 2017 Digital Globe, Image © 2017 CNES/Airbus). Flow direction is from top to bottom in all diagrams and images.

In the Australian drylands, a continuum of dominantly alluvial river types extends from relatively high-energy rivers that maintain a single, continuous channel downstream, to lower energy, declining or discontinuous rivers that undergo various forms of channel breakdown, including disintegration into networks of multiple, smaller channels and/or termination on unchannelled plains termed floodouts [cf.^17^ Fig. 1]. In some instances, channel breakdown is associated with extensive floodplain wetlands [e.g.^18^] that provide ecosystem services in these water-stressed settings (e.g. wildlife habitat, water filtration and supply). Globally, other dryland river types occur, including dominantly alluvial (e.g. braided), dominantly bedrock (e.g. incised or ingrown meanders), and mixed bedrock-alluvial (e.g. anabranching/anastomosing) types^19,20^. In this study, these river types have not been considered, as Australian dryland rivers are not braided and are dominantly alluvial in their middle and lower reaches. Previous research has suggested that regional continua of dryland river types may be related to hydroclimatic gradients, with greater aridity leading to a greater propensity for channel breakdown^21,22^. To refine these concepts and identify thresholds of change relevant for Australian dryland rivers, we characterise the hydrology and geomorphology of 29 rivers draining >1,800,000 km^2^ of continental eastern and central Australia, and establish the hydroclimatic conditions under which different river types persist. First, we categorise Australian dryland rivers into five types using a suite of geomorphological characteristics. Second, we establish the distinct hydrological characteristics of these river types using streamflow gauge data and correlate these characteristics with catchment aridity. Third, by establishing robust and significant relationships between modern dryland river types, hydrology and catchment aridity, we define envelopes of mean catchment aridity index (AI) values for each river type. Fourth, we use downscaled global climate model projections to project future hydroclimatic changes and associated geomorphological responses. Where projections suggest that a catchment will shift outside of the envelope of aridity defined by modern climatic data, we anticipate the trajectory of geomorphological change for the trunk river in that catchment and identify the geomorphological thresholds that may be crossed. With further refinement to include a wider range of dryland river types (e.g. mixed bedrock-alluvial types), this approach will provide a quantitatively-tested method for broader, continental and global studies of dryland river sensitivity to hydroclimatic changes that may be exacerbated or compounded by other human activities, such as future river regulation, flow diversion and abstraction, and land use change. Early identification of dryland river responses to global hydroclimatic changes will have far-reaching implications for the management of dryland rivers and the maintenance of dryland river ecosystem service delivery.

## Diversity of Australian dryland rivers

Much of continental eastern Australia is characterised by large catchments that host perennial, intermittent or ephemeral rivers and associated wetlands. These catchments are located in a post orogenic, intracratonic setting, where denudation rates and river sediment loads are low compared to global averages. Beyond the headwaters, many rivers follow lengthy courses across piedmonts and lowland plains where bedrock outcrop is less common and alluvial river styles dominate. Owing to limited tributary inputs and declining valley slopes, many rivers undergo downstream decreases in discharge and stream power that lead to channel size declines and, in some cases, channel breakdown^23^. Some of these rivers are regulated to allow water storage, abstractions and altered seasonal flows, but downstream decreases in discharge and stream power, and channel size declines and breakdown can occur despite regulation^23^. While Australian dryland rivers display a range of dominantly alluvial styles (e.g. single channel versus multiple channel), we have defined five river types that are widely represented in the middle to lower reaches (Fig. 1; Supplementary Fig. S1). Two overarching categories are recognised: rivers with through-going channels to their catchment outlet (e.g. a lake or another river) and rivers with discontinuous channels (e.g. characterised by zones where channels lose definition or where channels terminate). Further differentiation is achieved by defining dominant river planform (sinuous, non-sinuous) and pattern (meandering, straight, anabranching/anastomosing, distributary), the nature of downstream decline in channel size after leaving valley (bedrock) confinement (maintaining, declining, or terminating), and the presence/absence and type of wetlands (see Methods, Table 1 and Supplementary Fig. S1). The five types are: Type 1 – through-going, sinuous channels that maintain size downstream; Type 2 – through-going, sinuous channels that decline in size downstream; Type 3 – through-going to discontinuous, sinuous channels that decline in size downstream; Type 4 – discontinuous, non-sinuous channels that decline in size downstream; Type 5 – discontinuous, non-sinuous channels that decline in size downstream and terminate (e.g. at a floodout) (Fig. 1).

**Table 1:** Geomorphological measures used to differentiate the five dryland river types.

Channel continuity		Dominant river planform	Dominant river pattern	Wetlands (presence/ absence, type)	River type
Through-going	Maintaining	sinuous	meandering or anabranching/anastomosing	permanent wetlands	Type 1 – through-going, maintaining
	Declining			permanent and intermittent wetlands	Type 2 – through-going, declining
					Type 3 – through-going to discontinuous, declining
Discontinuous			meandering/straight, anabranching/anastomosing, or distributary		
		non-sinuous		intermittent and ephemeral wetlands	Type 4 – discontinuous, declining
	Terminating		straight, anabranching/anastomosing, or distributary, with trunk channel terminating in a floodout (unchannelled plain)	no significant wetlands	Type 5 – discontinuous, terminating

## Climatic gradient in continental eastern Australia

**Figure 2:**
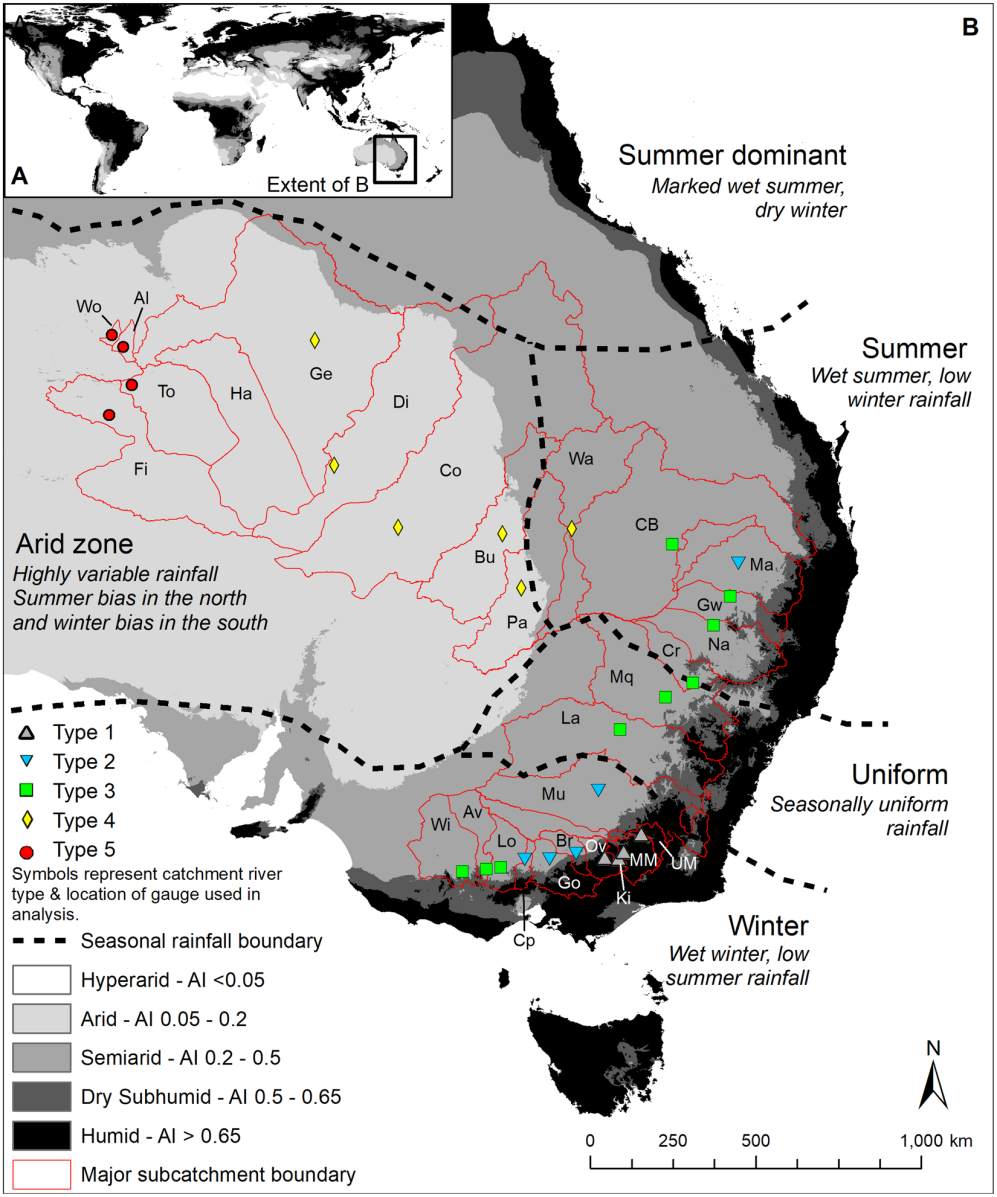
Modern, global Aridity Index [AI] (defined as mean annual precipitation [PPT]/annual potential evapotranspiration [PET]) for (**A**) the world and (**B**) central and eastern Australia. AI data were sourced from a publicly available dataset^57^. Drylands have AI < 0.65^56^. Currently, there are no hyperarid regions on the Australian continent. In B), seasonal rainfall boundaries are marked by dashed black lines and are adapted from the Australian Bureau of Meteorology’s climate classification data. Stream gauges were selected nearest to the end of bedrock confinement in each catchment and symbols represent different river types (see Fig. 1). Study catchments are labelled as follows: Ki – Kiewa, MM – Mitta Mitta, Ov – Ovens and UM – Upper Murray (Type 1; grey triangles); Br – Broken, Cp – Campaspe, Go – Goulburn, Ma – Macintyre and Mu – Murrumbidgee (Type 2; inverted blue triangles); Av – Avoca, CB – Condamine-Balonne, Cr – Castlereagh, Gw – Gwydir, La – Lachlan, Lo – Loddon, Mq – Macquarie, Na – Namoi and Wi – Wimmera (Type 3; green squares); Bu – Bulloo, Co – Cooper Creek, Di – Diamantina, Ge – Georgina, Pa – Paroo and Wa – Warrego (Type 4; yellow diamonds); Al – Allungra Creek, Fi – Finke, Ha – Hay, To – Todd and Wo – Woodforde (Type 5; red circles). See Table 3 for a list of all rivers. Note that there is no useable gauge in the Hay catchment. Map created in ArcMap v.10.2 software (https://desktop.arcgis.com/en/arcmap/).

Approximately 78% of the Australian continent is classified as dryland^24^, and across the eastern half of the continent, there is a pronounced aridity gradient from humid (AI > 0.65), through dry subhumid (AI 0.65–0.5) and semiarid (AI 0.5–0.2), to arid (AI 0.2–0.05) (Fig. 2). Pronounced periods of above- and below-average rainfall combine to also make Australian hydroclimates (including river flow regimes) some of the most variable in the world^25–29^, a characteristic that persists despite the presence of dams and associated flow regulation in many catchments. In this tectonically stable setting, and especially in the middle to lower reaches where rivers are largely free from bedrock influence, these hydroclimatic variations are the key driver of river response and resulting channel-floodplain geomorphology. The relationship between climate, hydrology, and geomorphology is complex but may be expressed through various metrics including mean and peak runoff depth, flow variability, and stream power. Stream power is a function of river discharge and slope, and represents the energy exerted by water on the bed and banks of a river^30^. It is widely used as a quantitative measure of the potential for flow to initiate channel adjustment through erosion or deposition^31,32^.

## Hydroclimatic controls on Australian dryland river geomorphology

**Figure 3:**
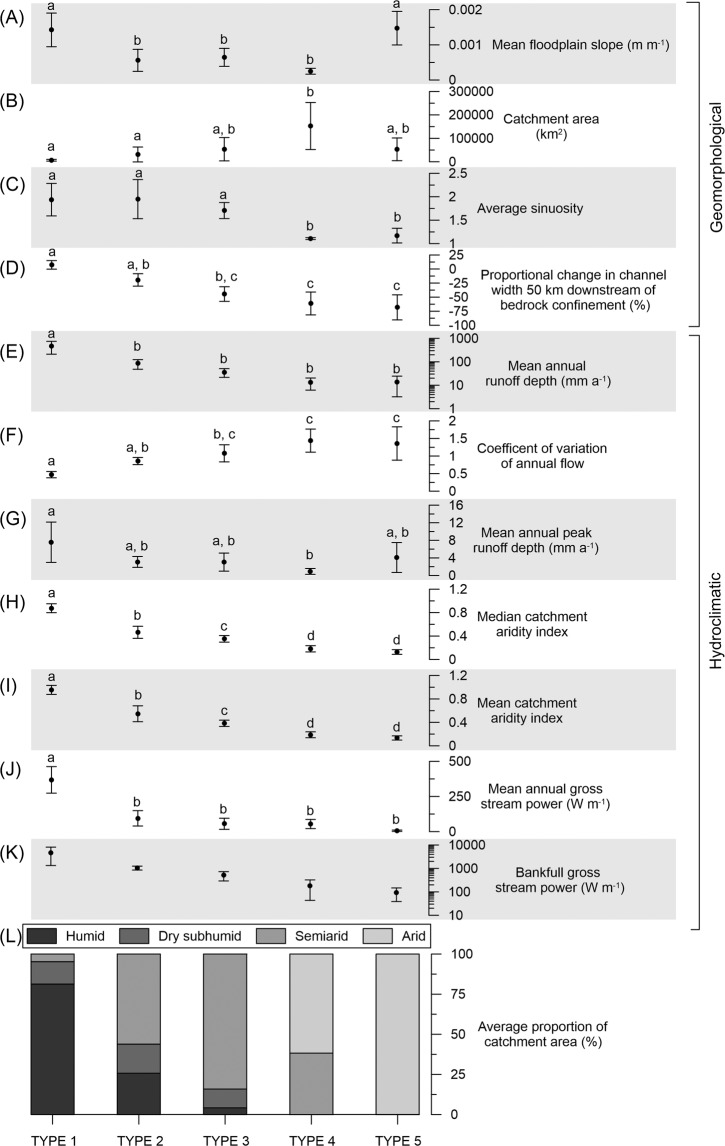
Comparison of the five river types based on 11 geomorphological and hydroclimatic variables. Plots show mean plus error bars representing one standard deviation (Type 1, n = 4; Type 2, n = 5; Type 3, n = 9; Type 4, n = 6; Type 5, n = 4). One-way ANOVAs were performed to identify statistically significant differences between river types for each variable. Points sharing the same letter are not significantly different according to Tukey’s honest significant difference (HSD) test. (**A**) No systematic trend between floodplain slope and river type. (**B**) No systematic trend between catchment area and river type, although Types 1 and 2 tend to have smaller catchments. (**C**) Significant differences between sinuosity and river type, with Types 1, 2, and 3 having significantly higher sinuosity than Types 4 and 5. (**D**) Negative correlation between rate of downstream change in channel size and river type. (**E**) Negative correlation between mean annual runoff depth and river type, although the only significant difference is between Type 1 and the other types. (**F**) Positive correlation between CV_af_ and river type, albeit with substantial overlap between types. (**G**) Weak negative correlation between mean annual peak runoff depth and river type. (**H**) Significant differences between river types explained by median catchment Aridity Index (AI), except for Types 4 and 5. (**I**) Significant differences between river types explained by mean catchment AI. Mean catchment aridity is the best variable to differentiate river types, except for Types 4 and 5. (**J**) Negative correlation between mean annual gross stream power (measured at gauge nearest to the end of bedrock confinement) and river types. (**K**) Negative correlation between bankfull gross stream power (measured at gauge nearest to the end of bedrock confinement) and river type, although there are not enough gauge data available to perform one-way ANOVAs. (**L**) Average proportion of catchment in each climate zone for each of the five river types, demonstrating the progressive increase in the overall level of catchment aridity from Type 1 to Type 5.

**Figure 4:**
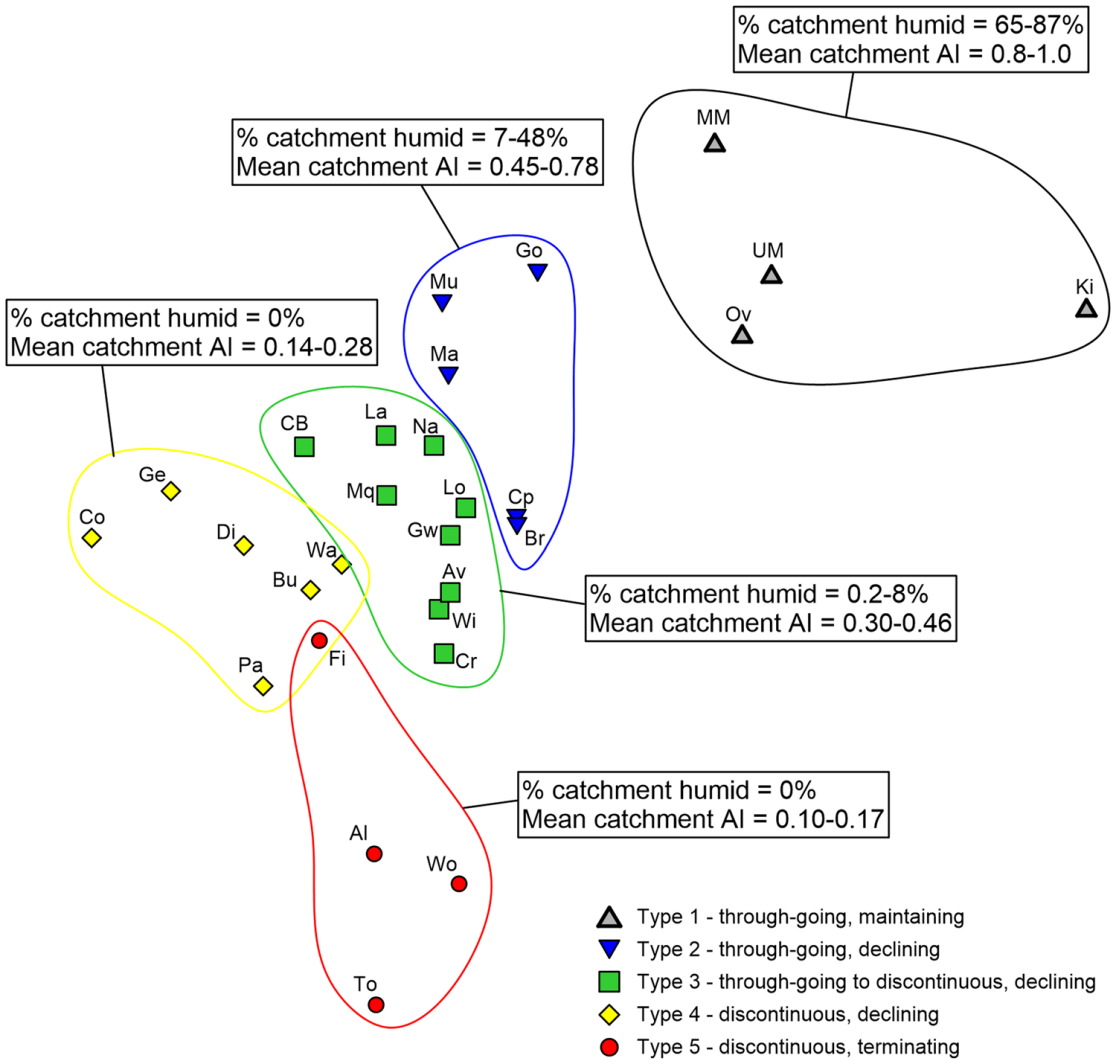
Non-parametric multidimensional scaling (nMDS) resemblance matrix visualising the level of similarity between each river type based on key geomorphological and hydroclimatic variables (see Methods; Table 2). Pairwise ANOSIM analysis shows that each river type is statistically different from one another (*p* < 0.05; see Table 2 and Supplementary Table S1). Each point on the nMDS plot represents a different river and points closer together are more similar to one another based on the measured variables, and points further apart are more dissimilar. Annotations summarise the percentage of catchment area classed as humid for each river type, and the envelopes of mean catchment AI values for each river type. See Fig. 1 for catchment labelling conventions and Table 3 for a list of all rivers.

To examine the links between hydroclimate and geomorphology, the five river types (Fig. 1; Table 1) have been subjected to one-way analysis of variance (ANOVA; Fig. 3) and a pairwise analysis of similarities (ANOSIM) of key variables (Fig. 4; Table 2; Supplementary Table S1). Mean catchment AI is the variable that best defines significant differences between river types (except for Types 4 and 5; see Fig. 3I). Through-going, maintaining rivers (Type 1) occur in catchments where ~65–87% of the total catchment area is classed as humid and mean catchment AI is 0.80–1.0 (Fig. 4; Supplementary Fig. S2). Through-going, declining rivers (Type 2) occur in catchments where ~7–48% of total catchment area is classed as humid and mean catchment AI is 0.45–0.78. Through-going to discontinuous, declining rivers (Type 3) occur in catchments where ~0.2–8% of the total catchment area is classed as humid and mean catchment AI is 0.30–0.46. Discontinuous, declining rivers (Type 4) have no catchment area that is humid or even dry subhumid and mean catchment AI is 0.14–0.28. Discontinuous, terminating rivers (Type 5) occur in catchments that are typically 100% arid and mean catchment AI is 0.10–0.17 (Fig. 4; Supplementary Fig. S2).

**Table 2:** Variables used in ANOSIM and their units.

Variable	Units
Catchment area	km^2^
Average slope of alluvial plain	m m^−1^
Average sinuosity (channel distance/ valley distance)	Dimensionless
Proportional net change in channel width 50 km downstream of confinement	(%/50 km downstream of bedrock confinement)
Mean annual runoff depth	mm a^−1^
Mean annual peak runoff depth	mm a^−1^
Coefficient of variability of annual flow (std. dev./mean)	%
Mean annual gross stream power	W m^-1^
*Bankfull gross stream power	W m^-1^
Mean catchment AI (mean annual precipitation/potential annual evapotranspiration)	Dimensionless
Median catchment AI (mean annual precipitation/potential annual evapotranspiration)	Dimensionless

Geomorphological measures were calculated using Google Earth satellite imagery and a 30 m SRTM DEM. (Note: * not used in statistical analysis).

**Figure 5:**
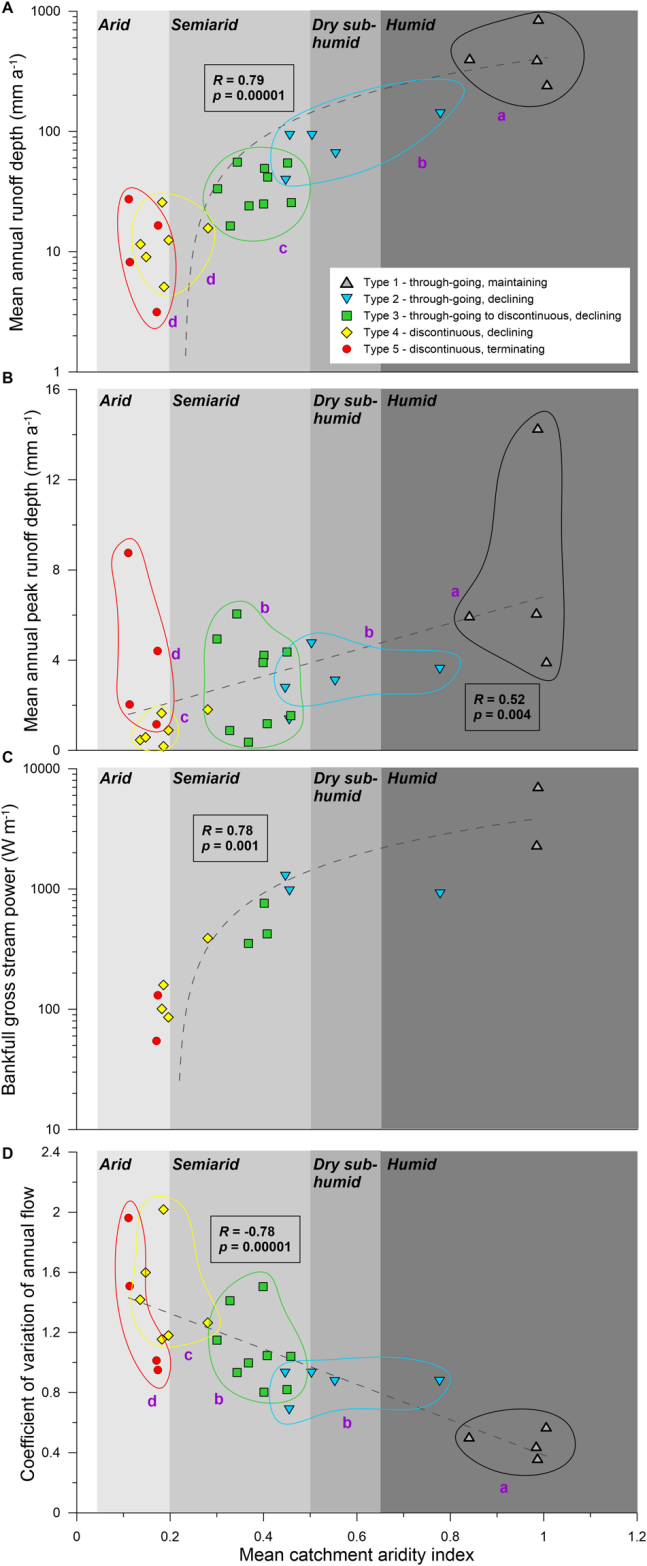
Mean catchment Aridity Index (AI) plotted against various hydrologic parameters from river gauge locations nearest to the end of bedrock confinement in each catchment (see Fig. 2 for gauge locations). Pearson’s correlation coefficient (*R*) indicates the strength of the linear association between the variables (note that the log-scale for A and C makes the regressions appear curved). The p-value (*p*) indicates the statistical significance of the linear correlation between the variables. Lowercase purple letters indicate the results of a pairwise ANOSIM; groups sharing the same letter are not significantly different. (**A**) mean annual runoff depth (mm a^−1^); (**B**) mean annual peak runoff depth (mm a^−1^); (**C**) bankfull gross stream power (estimated using bankfull discharge estimates from gauged cross sections and ratings tables). In this case, there are insufficient gauge data to confidently estimate bankfull stream power for all rivers, meaning statistical analysis across river types cannot be undertaken; (**D**) Coefficient of variation of annual flow (CV_af_).

Links between climate, catchment aridity, and key hydrological variables have been established in previous research, including how the magnitude and variability of runoff and streamflow in eastern Australian rivers is strongly modulated by climatic modes (e.g. El Niño-Southern Oscillation (ENSO), Interdecadal Pacific Oscillation (IPO) and the Southern Annular Mode (SAM))^25–29^. While these inter- and multi-decadal hydroclimatic relationships are relatively well understood, the distinct trends and clusters that emerge from our analysis demonstrate, for the first time, the significant overarching control that dominant hydroclimate exerts on the geomorphology of Australian dryland rivers. There is a strong and significant positive correlation between mean catchment AI and mean annual runoff depth (Pearson’s *R* = 0.79, *p* = 0.00001), and all river types are significantly different except for Types 4 and 5 (pairwise ANOSIM, *p* < 0.05; Fig. 5A). There is a significant positive correlation between mean catchment AI and mean peak annual runoff depth (Pearson’s *R* = 0.59, *p* = 0.004), and all river types are significantly different except for Types 2 and 3 (pairwise ANOSIM, *p* < 0.05; Fig. 5B). There is a strong and significant positive correlation between mean catchment AI and bankfull gross stream power (Pearson’s *R* = 0.78, *p* = 0.001; Fig. 5C). There is a strong and significant negative correlation between mean catchment AI and coefficient of annual flow (CV_af_; Pearson’s *R* = −0.78, *p* = 0.00001), and all river types are significantly different except for Types 2 and 3 (pairwise ANOSIM, *p* < 0.05; Fig. 5D).

Overall, catchments with a mean catchment AI classed as humid or dry subhumid have greater mean annual runoff depth (>40 mm a^−1^) and stream power (>900 W m^−1^), lower flow variability (CV_af_ < 0.9), and support through-going river types (most Types 1 and 2). Catchments with a mean catchment AI classed as semiarid or arid have lower mean annual runoff depth (<55 mm a^−1^) and gross stream power (<760 W m^−1^), greater flow variability (CV_af_ > 0.8), and tend to be characterised by discontinuous river types (most Type 3 rivers, and Types 4 and 5; Fig. 5). These metrics enable the identification of thresholds that define the hydroclimatic conditions that are responsible for channel (dis)continuity in these dryland rivers (i.e. through-going vs. discontinuous). There is also an intrinsic, but poorly defined, channel termination threshold causing Type 4 and 5 rivers to break down into unchannelised wetlands or floodouts.

These findings highlight the key influence of hydroclimatic conditions on the river types across our identified continuum, manifested in differences in river planform, pattern and downstream channel (dis)continuity (Fig. 1). Many Australian dryland rivers, especially Types 2, 3, 4 and 5, are characterised by downstream declines in bankfull discharge (Supplementary Fig. S1), which result from flow transmission losses associated with floodwave attenuation, evapotranspiration, or groundwater recharge^23,33,34^. Downstream declines in bankfull discharge are commonly associated with downstream decreases in channel slope, which collectively result in downstream decreases in stream power and sediment transport capacity, thus promoting sediment deposition. These changes may lead to a contraction of channel width and/or to channel avulsion, the process of channel relocation on a floodplain [e.g.^35^]. Avulsion can form new channels that may supersede the original trunk channel in single-channel rivers, or create new channels that operate in parallel with the trunk channel in multi-channelled rivers. In more arid settings, however, there is a threshold beyond which stream power becomes too low to maintain sediment transport in a defined channel. Hence, channel breakdown occurs, leading to unchannelised wetlands (Types 3 and 4) and/or floodouts without significant wetlands (Type 5), particularly where there are barriers to flow resulting from aeolian or alluvial deposits, or local bedrock outcrop^17^.

Recognition of these links between hydroclimates and dryland river geomorphology allows each of the five river types to be defined within a range of mean catchment AI values. Establishing these AI envelopes allows us to identify which rivers exist close to a threshold between different river types, and then use climate projections to forecast future trajectories of change for rivers subject to changing hydroclimatic conditions.

## Climate change projections and impacts on Australian dryland rivers

**Figure 6:**
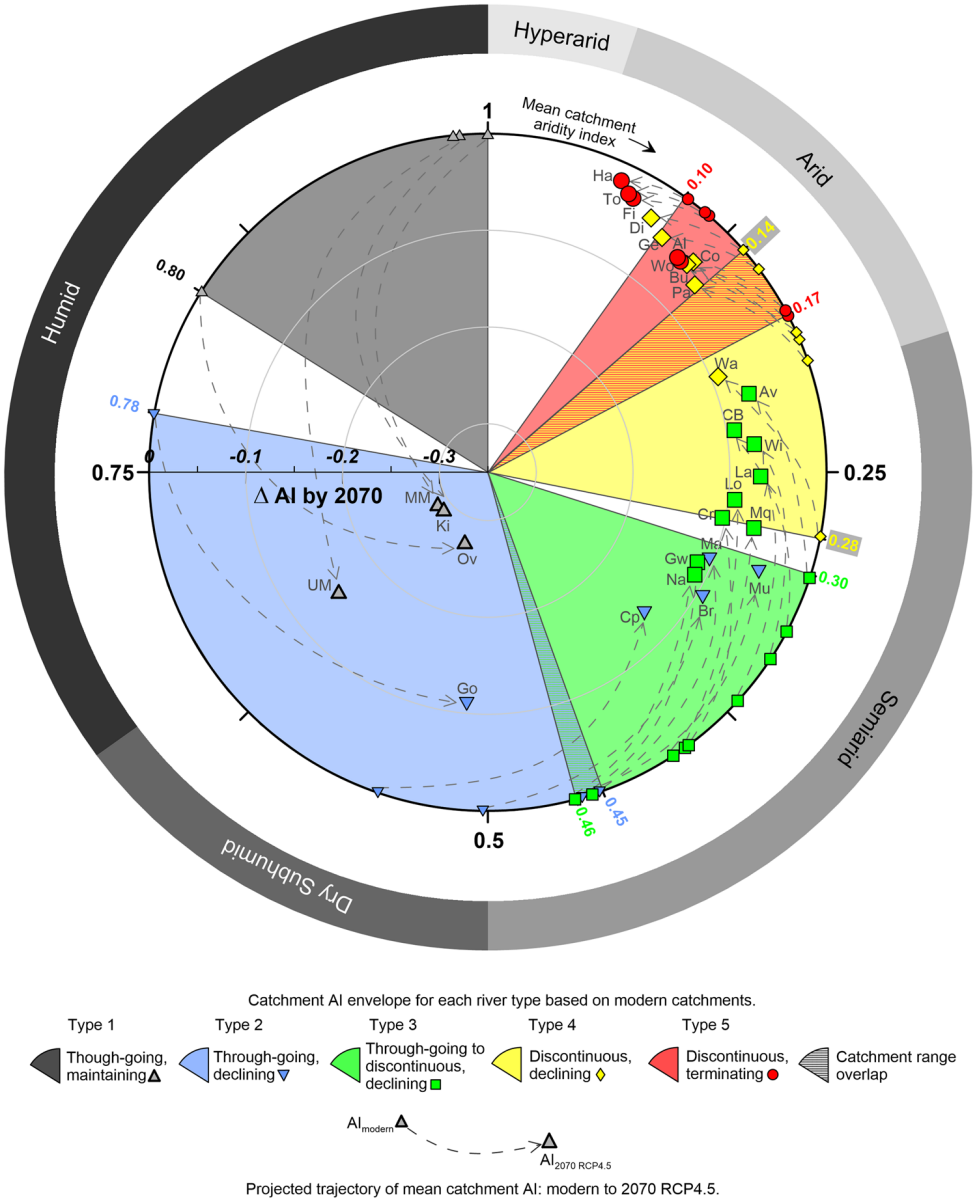
Polar plot displaying the modern and projected mean catchment Aridity Index (AI) value for each catchment (angle axis) and the projected magnitude of change in the mean catchment Aridity Index by 2070 RCP4.5 (radius axis; Δ AI by 2070). Dashed arrowed lines represent trajectories that connect the starting (modern) and end (2070) points for individual rivers; rivers that end closest to the centre of the plot have the largest projected Δ AI. Coloured segments represent the envelope of AI values assigned to each river type based on modern catchment characteristics. Symbols represent the modern river type and those that are a different colour to the underlying segment are rivers that are projected to change river type by 2070 according to RCP4.5. See Fig. 2 for catchment labelling conventions and Table 3 for a list of all rivers.

Previous studies investigating future hydrological changes to inland Australian rivers have predicted lower stream flows resulting from warming and altered precipitation patterns^36–39^. Decreases in runoff in southern Australia, resulting from both reductions in winter rainfall and increased temperatures, have been projected with a high confidence^38,39^. These projected runoff decreases in southern Australia accord with our projections of increased future aridity in continental eastern Australia for 2070 (see Supplementary Fig. S3) under Representative Concentration Pathway 4.5 (RCP4.5; [cf.^40^]). RCP4.5 is a relatively moderate climate change pathway and in our study was chosen to understand which rivers are the most sensitive to conservative scenarios of future climate change. Interestingly, our modelling of future aridity under RCP8.5 suggests very similar changes in aridity over the Australian continent (Supplementary Fig. S3), likely due to increased projected rainfall across parts of northern and central Australia under RCP8.5 as well as higher projected temperatures relative to RCP4.5, thus resulting in a similar net projected change in mean catchment AI for both scenarios. Since contemporary levels of catchment aridity are closely correlated with mean and peak annual runoff depth, stream power, and flow variability (Fig. 5), and different hydroclimatic conditions are associated with distinct river types (Figs. 3, 4 and 5), we can assess how Australian dryland rivers may respond to projected hydroclimatic changes related to changing aridity indices. Given the magnitude of rainfall reductions and evapotranspiration increases projected for much of the Australian continent under RCP4.5 (Supplementary Fig. S3), many catchments will likely experience substantial aridification (Fig. 6; Supplementary Figs. S2 and S3). Particularly sensitive rivers are those that are close to a threshold of change between different river types and that therefore may be subject to significant geomorphological adjustments under these future hydroclimatic conditions.

Indeed, of the 29 rivers analysed in this study, 23 (~80%) are projected to cross a threshold to another river type due to future catchment aridification and associated changes in hydrological conditions under RCP4.5 (Fig. 6). The largest magnitude changes are likely to affect the currently through-going, maintaining rivers (Type 1) with headwaters in the Australian southeastern highlands. These highlands are projected to have some of the most severe reductions in rainfall (see Supplementary Fig. S3), likely as a result of a projected increase in the prevalence of positive phases of the Southern Annular Mode, which will shift the average track of the southern hemisphere westerlies farther south and reduce winter rainfall over southern Australia^41^. All four Type 1 rivers are projected to change to through-going, declining rivers (Type 2). Reduced runoff depth and stream power will likely lead to sediment deposition and downstream declines in channel size (Fig. 6). Although the Kiewa, Mitta Mitta, Upper Murray and Ovens rivers (Type 1) occupy only a small proportion of the Murray-Darling Basin (~3.8%), they currently supply a disproportionately large amount of discharge (>33%) to parts of the lower catchment. Therefore, reductions in mean annual runoff depth and downstream declines in flow will have major impacts for water users and ecosystem services in the southern Murray-Darling Basin, which is one of Australia’s most important agricultural regions. While we have not modelled the effects of future water storage, flow diversions and abstractions, and land use in these catchments, these other forms of human disturbance will likely exacerbate or compound the effects of climate change by further altering flow regimes in already highly pressured, regulated systems^6^.

The most dramatic change in river morphology is from a through-going, declining river (Type 2) to a more discontinuous river (Types 3 or 4). Under RCP4.5, the currently through-going, declining Campaspe, Broken, Macintyre, and Murrumbidgee rivers (Type 2) are projected to have significant increases in catchment aridity, leading to reduced discharges and stream powers and more highly variable flow regimes, thereby becoming discontinuous rivers more characterised by channel breakdown and unchannelised wetlands (Type 3). Such dramatic changes in river structure and function will decrease hydrological and sediment connectivity, with profound, long-lasting impacts on water and sediment distribution, ecosystem dynamics, and ecosystem services in rivers and floodplains that are currently heavily used for irrigated agriculture and grazing. These changes are potentially irreversible and will increase the likelihood of ecosystem collapse in these marginal environments [c.f.^42^]. This will likely exacerbate existing high tensions surrounding water use and storage in inland Australia, particularly in the Murray-Darling Basin where ongoing drought (2018–2020) and water resource planning changes have had severe ecological impacts, perhaps providing an insight into the future facing many of Australia’s dryland rivers^43,44^.

All of the currently through-going to discontinuous (Type 3) rivers except the Gwydir and Namoi are projected to become Type 4 rivers with discontinuous, declining channels (Fig. 6), likely inducing changes to many of the semi-permanent, in-channel waterholes that currently characterise many river reaches^19^. Catchment aridification may also impact inundation regimes in semi-permanent and intermittent floodplain wetlands associated with these rivers, likely inducing a change to more ephemeral floodplain wetlands. Potential reductions in discharge and flow frequency will be particularly profound for wetlands of national and international ecological importance (e.g. Ramsar wetlands), such as the Macquarie Marshes, Gwydir wetlands, Narran Lake wetlands, and the Great Cumbung Swamp on the lower Lachlan River. Except for the Warrego River (Fig. 6), further declines in flow may even result in many Type 4 rivers transitioning into discontinuous, terminating rivers (Type 5) with few or no significant wetlands.

Discontinuous, terminating rivers (Type 5) are projected to experience even more arid hydroclimatic conditions, most likely with even greater flow variability than at present. While there are currently no Australian dryland rivers that are characterised by the levels of aridity projected for the Hay, Todd, and Finke catchments (Fig. 6), increased flow variability may impact severely on the health of the well-developed riparian vegetation assemblages that are such a critical influence on channel processes and forms in these rivers^19^. There are no hyperarid regions (AI < 0.05) currently on the Australian continent, but reference to other hyperarid areas around the world (e.g. central Sahara Desert), may provide an indication of what might be expected for the small regions of Australia projected to experience hyperarid conditions by the second half of this century (e.g. lack of bank-stabilising vegetation; Supplementary Fig. S3).

In summary, greater aridification of the Australian continent is projected for the second half of this century under both moderate (RCP4.5) and business-as-usual (RCP8.5) climate change scenarios. The influence of this enhanced aridification on the hydrology, physical structure, and biogeomorphic function of Australian dryland rivers is likely to be dramatic but, until now, has not been considered in any rigorous or systematic manner. Our approach is a quantitively-tested form of ergodic reasoning with powerful application for understanding river response to future hydroclimate change, not only across continental Australia but also potentially in other drylands worldwide, as discussed below.

## Developing the approach to assess river response in drylands globally

Our findings demonstrate that even under a relatively conservative future emissions scenario (RCP4.5), projected aridification and associated changes to hydrological conditions across continental eastern and central Australia are likely to lead to significant geomorphological thresholds being crossed this century. The vast majority of rivers included in this study were highly sensitive to hydroclimatic change.

While Australia possesses a number of distinctive river styles, none of these styles are necessarily unique to the continent [e.g.^19^]. Hence, with further development, the novel approach outlined in this study potentially could be used to assess the geomorphic sensitivity of dryland rivers globally, especially for dominantly alluvial rivers where hydroclimatic factors are the key drivers of river response. Many parts of dryland Africa, South and North America, and central and western Asia are generally projected to experience declines in surface water availability and river flow in coming decades^6,13,14,45–48^. As such, rivers in these regions may also respond to these hydroclimatic changes by transforming from through-going to more discontinuous river types. In southern Africa, for instance, river morphology varies across strong hydroclimatic gradients, with through-going, maintaining rivers more common in subhumid catchments, and through-going, declining and discontinuous, declining or terminating rivers more common in semiarid and arid catchments^21,22^. Nevertheless, along many of these rivers, varying degrees of bedrock (lithological, structural) control influence the river’s ‘degrees of freedom’; for example, resistant rocks such as dolerite or quartzite commonly crop out in river bed and banks and influence the depth of incision or extent of lateral migration that can occur in response to hydroclimatic drivers [e.g.^49,50^]. In drylands where there is an even higher degree of tectonic and bedrock influence on rivers (e.g. the Mediterranean and parts of the Middle East), 21^st^ century hydroclimatic drivers will still exert an influence on river response, but may be muted or locally overridden by the non-climatic factors. For example, in steep, confined river valleys, dryland rivers may respond to hydroclimatic changes mainly by altering the magnitude of cut-and-fill cycles rather than by undergoing any pronounced changes in channel continuity while periodic, tectonically-induced rockfalls or landslides may provide the main reach-scale, long-lasting disruptions to downstream flow and sediment transfer^19,51^. In many regions globally, river regulation and flow abstraction can also impact on the flow regime of dryland rivers^6,43^, although in most cases the effect would be similar to that expected under projected future hydroclimatic change (i.e. reduced runoff and perhaps altered seasonality) and so will likely exacerbate or compound the hydrological and geomorphological changes brought about by future hydroclimate change.

While our approach has been based around Australian dryland rivers with flow gauging records, this may not be essential. Climate-hydrology-geomorphology relations established for gauged rivers could be used to make predictions for nearby ungauged rivers in the same hydroclimatic region. In southeastern Australia, for example, all the gauged Type 1 rivers that form part of our dataset (e.g. Kiewa, Mitta Mitta, Upper Murray and Ovens rivers) are projected to become Type 2 rivers under future hydroclimatic change (Fig. 6). Modelling of future mean catchment aridity index for other nearby rivers that are classified as Type 1 based on their geomorphology can be undertaken even if they are ungauged, as the robust climate-hydrology-geomorphology relationships have been established using data from the gauged rivers. Alternatively, indirect approaches to discharge estimation in dryland rivers^52^ could be used to generate supporting hydrological or hydraulic information to help establish modern climate-hydrology-geomorphology relations across hydroclimatic gradients, as has been done successfully for different dryland rivers worldwide (e.g.^10,22,53^). By applying ergodic reasoning, these relations could then be used as the basis for predicting river response under altered hydroclimates.

In summary, beyond the continental Australian setting, our approach will need to be expanded to include a wider diversity of dryland rivers (e.g. additional alluvial, bedrock, and mixed-bedrock alluvial types), and to account for a fuller suite of climatic and non-climatic (e.g. lithological/structural, tectonic, anthropogenic) controls on river response. Nevertheless, careful regional- or continental-scale application to a range of gauged and ungauged dryland rivers may still provide the basis for making valuable insights into potential threshold river responses to 21^st^ century hydroclimatic changes.

### Conclusion

Forecasting the responses of dryland rivers to climate-driven hydrological changes is critical due to the importance of geomorphology for the range and quality of ecosystem services in these marginal environments^1^. Dryland rivers sustain many unique and threatened ecosystems and help support the livelihoods of approximately 2 billion people who live in the world’s drylands^1,2^. Given heightened concern over the likelihood of a global average temperature rise of >1.5 °C by the end of the century^54,55^, major changes to hydroclimates and river geomorphology are likely to occur within a few generations. Our approach to forecasting likely geomorphological changes in Australian dryland rivers has the potential to be applied to other gauged and ungauged dryland rivers globally, including in regions where there is a wider diversity of river styles and additional controls that may influence river response to hydroclimatic changes. As such, further development of our approach may provide a key tool to identify thresholds of river response in otherwise poorly monitored systems, and may provide advance warning of such changes to inform water resource development, adaptation, and other management strategies.

## Methods

### River types

Large river catchments in continental eastern and central Australia straddle different climate zones from humid through to arid (Fig. 2). The rivers included in this study in some cases have humid upper reaches, but all have drylands (subhumid through arid climates) consisting of between 10 and 100% of their catchment areas. A framework was developed to categorise river types based on their geomorphological characteristics. The rivers analysed were the Kiewa, Mitta Mitta, Ovens, Upper Murray, Broken, Campaspe, Goulburn, Macintyre, Murrumbidgee, Avoca, Condamine-Balonne, Castlereagh, Gwydir, Lachlan, Loddon, Macquarie, Namoi, Wimmera, Bulloo, Cooper Creek, Diamantina, Georgina, Paroo, Warrego, Allungra Creek, Finke, Hay, Todd, and Woodforde. Although dryland river types fall along a continuum^19,20^, we use five geomorphological characteristics to define five dryland river types (Fig. 1; Table 1; see also Supplementary Fig. S1). These geomorphological characteristics were derived using Google Earth satellite imagery and a 30 m Shuttle Radar Topography Mission (SRTM) digital elevation model (DEM):

1. longitudinal continuity of channels (through-going, discontinuous);
2. nature of reduction in channel width downstream of the end of major bedrock confinement (maintaining, declining, terminating);
3. dominant river planform (sinuous, non-sinuous);
4. dominant river pattern (single-thread (meandering, straight), multi-thread (anabranching, anastomosing, distributary));
5. presence/absence of wetlands, and where present, type of wetlands (e.g. permanent, intermittent, ephemeral).

### Aridity and hydrology

Aridity has been expressed according to the Aridity Index (AI), defined as mean annual precipitation (PPT) divided by potential mean annual evapotranspiration (PET^56^). AI data were sourced from the datasets compiled by Trabucco and Zomer, (2009^57^), available from the Consultative Group for International Agriculture Research Consortium for Spatial Information (CGIAR-CSI) GeoPortal (http://www.csi.cgiar.org). Hydrology data were sourced from the New South Wales Department of Primary Industries Office of Water, the Victorian Department of Environment, Land, Water and Planning Water Measurement Information System, the Queensland Government Water Monitoring Information Portal, and the Northern Territory Water Resources Data and Information Centre. Gauges were chosen as near as possible to the end of bedrock confinement on each river (i.e. usually in the transition from the upper to middle reaches) and each had at least 30 years of continuous flow records, except for the Finke River and Allungra Creek gauges that had 13 and 16 years of flow records, respectively (Table 3). It should also be noted that the Hay River does not have a suitable gauge (the only gauges in the catchment are on small headwater creeks) and was not included in hydrological analysis, but was included for catchment aridity analyses.

**Table 3:** Rivers with stream gauges (n = 28) used for hydrological analysis in this study (see Fig. 2). The Hay River does not have a suitable gauge.

River	River code	Gauge name	Gauge number	Period of record	Number of years
Mitta Mitta	MM	Tallandoon	401204	1934–2017	83
Kiewa	Ki	Mongan’s Bridge	402203	1955–2017	62
Upper Murray	UM	Jingellic	401201	1900–2017	117
Ovens	Ov	Myrtleford	403210	1961–2017	56
Goulburn	Go	Murchison	405200	1900–2017	117
Campaspe	Cp	Barnadown	406201	1978–2017	39
Broken	Br	Casey’s Weir	404242	1972–2017	45
Murrumbidgee	Mu	Narrandera	410005	1914–2017	103
Macintyre	Ma	Boggabilla	416002	1900–2017	117
Lachlan	La	Condobolin	412006	1900–2017	117
Macquarie	Mq	Baroona	421127	1986–2017	31
Gwydir	Gw	Pallamallawa	418001	1900–2017	117
Namoi	Na	Mollee	419039	1965–2017	52
Loddon	Lo	Laanecoorie	407203	1900–2017	117
Wimmera	Wi	Eversley	415207	1963–2017	54
Condamine-Balonne	CB	St George	422201F	1971–2017	46
Avoca	Av	Archdale Junction	408206	1987–2017	30
Castlereagh	Cr	Mendooran	420004	1953–2010	57
Warrego	Wa	Wyandra	423206A	1967–2017	50
Paroo	Pa	Willara Crossing	424002	1975–2017	42
Cooper Creek	Co	Nappa Merrie	003103A	1965–2017	52
Bulloo	Bu	Autumnvale	011202A	1967–2017	50
Diamantina	Di	Birdsville	A0020101	1966–2017	51
Georgina	Ge	Roxborough Downs	001203A	1967–2017	50
Finke	Fi	Finke RS Crossing	G0050116	2004–2017	13
Todd	To	Heavitree Gap	G0060126	1973–2017	44
Woodforde	Wo	Arden Soak	G0280010	1975–2017	42
Allungra Creek	Al	Allungra Waterhole	G0280004	2001–2017	16

Hydrological variables were calculated from these flow records using standard methods. Mean annual runoff depth was calculated by dividing mean annual discharge (megalitres; ML) by catchment area (km^2^). Mean annual peak runoff depth was calculated by dividing mean annual peak discharge (ML) by catchment area (km^2^). Coefficient of variation of annual flow (CV_af_) was calculated by dividing the standard deviation of mean annual discharge by mean annual discharge. Mean floodplain slope was derived by calculating an average slope of the alluvial plain downstream of each river’s emergence from bedrock confinement using a 30 m SRTM DEM. Gross bankfull stream power is the product of bankfull discharge (m^3^ s^−1^), channel slope (m m^−1^), and the specific weight of water-sediment mixture (given the low suspended loads of most Australian rivers, this was assumed constant at 9800 N m^-3^). Gross bankfull stream power was calculated for the trunk channel at gauge locations that provided a ratings table necessary to estimate bankfull discharge; these data were only available for 14 of the 29 rivers and so there were not enough data points from each of the river types for robust analysis of similarities (ANOSIM) (see below and Fig. 5C). Channel slope data used for gross stream power estimation were calculated by dividing the local floodplain slope – calculated using 3–5 km transects along the floodplain at each gauge location using a 30 m SRTM DEM – by the channel sinuosity value measured over the same reach. The relationships between aridity and specific hydrological variables (mean annual runoff depth [annual discharge normalised for catchment area], mean annual peak runoff depth, and coefficient of variation of annual flow) were then analysed using basic linear regressions.

### Climate data and future climate projections

ArcMap v.10.2 software was used for catchment scale analyses of AI, precipitation, and future climate model outputs. Gridded modern AI data were clipped to each study subcatchment and the proportional aridity was calculated with exported cell counts of each unique AI value in the sub-catchment (cells equivalent to ~1 km by ~1 km). Weighted mean catchment AI was also calculated from this output.

To derive future precipitation and potential evapotranspiration, we used outputs from 16 downscaled global climate models (GCMs) that were part of the Intergovernmental Panel on Climate Change’s (IPCC) Fifth Coupled Model Intercomparison Project, (CMIP5^58^). These models are BCC-CSM1–1, CCSM4, CNRM-CM5, GFDL-CM3, GISS-E2-R, HadGEM2-AO, HadGEM2-CC, HadGEM2-ES, INMCM4, IPSL-CM5A-LR, MIROC-ESM-CHEM, MIROC-ESM, MIROC5, MPI-ESM-LR, MRI-CGCM3, and NorESM1-M. The outputs used were from model runs assuming Representative Concentration Pathway (RCP) 4.5. RCPs are named according to the radiative forcing of greenhouse gases expected for 2100 depending on various scales of emissions reductions (RCP2.6, 4.5, 6, 8.5)^40^. RCP4.5 is a relatively moderate future pathway in which emissions peak around 2040 and then decline with a radiative forcing of 4.5 W m^−2^ above pre-industrial values by 2100^40^. RCP4.5 was chosen to understand which dryland river catchments were most sensitive to even relatively moderate scenarios of future climate change. Future aridity under RCP8.5 was also modelled to understand the difference between scenarios (see Supplementary Fig. S3). Due to projected increases in rainfall over parts of northern and central Australia under RCP8.5 relative to RCP4.5, projected net change in AI over the Australian continent is very similar for both RCP4.5 and RCP8.5 (Supplementary Fig. S3). Future projected precipitation is an output from the CMIP5 models. Mean annual temperature (Tmean) and annual temperature range (Trange) were the outputs used in conjunction with solar radiation (RA) to model future potential evapotranspiration (PET) using the Hargreaves-Samani equation (Eq. (1)^59–61^):

1 $$PET=0.0023RA(Tmean+17.8)Trang{e}^{0.5}$$

The Hargreaves-Samani equation was used to calculate the modern AI used in this study^57^ and therefore was also used for the future projections. It has been shown to perform nearly as well as the common FAO Penman method but requires substantially less parametrisation with more readily available data^62^. Future projected precipitation was then divided by modelled future potential evapotranspiration to provide future AI values across Australia.

### Multivariate analysis of variables associated with river types

The five river types were compared across various hydrological and geomorphological variables using a multivariate approach in Primer v6^63^. Data for 11 variables were collected (Table 2; Figs. 3 and 4), but bankfull gross stream power was not included in statistical tests as data were not available to calculate gross stream power for all gauges. One-way Analysis of Variance (ANOVA) tests were also performed to determine the difference between river types for each variable. Tukey’s honest significant difference (HSD) test determined which groups were significantly different from one another at *p* < 0.05. Results are presented in Fig. 3. Analysis of similarities (ANOSIM) and pairwise tests among all combinations of river types were performed on a resemblance matrix based on Euclidean distances, and based on 9999 permutations. R statistics were calculated for each pairwise comparison, where R values closer to 1 indicate greater differences between two compared groups^63^. Significance was determined where *p* < 0.05 (Supplementary Table S1). Based on the resemblance matrix, a non-parametric multidimensional scaling (nMDS) plot was constructed to visually represent the similarity among rivers; each point on the figure represents a single river, with points closer together being more similar to one another on the measured variables, and points farther apart being more dissimilar (Fig. 4).

## Supplementary information

Supplementary Information.

## Data Availability

Modern aridity index data are publicly available from Trabucco and Zomer’s (2009) Global Aridity Index (Global-Aridity) and Global Potential Evapo-Transpiration (Global-PET) Geospatial Database. Published online, available from the CGIAR-CSI GeoPortal at: http://www.csi.cgiar.org. Downscaled global climate model projection data are publicly available from WorldClim – Global Climate data at: www.worldclim.org/. The data that support the findings of this paper are available from the corresponding author upon reasonable request.
