# Author Correction: Identifying threshold responses of Australian dryland rivers to future hydroclimatic change

**DOI:** 10.1038/s41598-020-69597-5

**Published:** 2020-07-22

**Authors:** Z. T. Larkin, T. J. Ralph, S. Tooth, K. A. Fryirs, A. J. R. Carthey

**Affiliations:** 1 Department of Earth and Environmental Sciences, Macquarie University, North Ryde, NSW 2109 Australia; 2 Department of Geography and Earth Sciences, Aberystwyth University, Aberystwyth, SY23 3DB UK; 3 Department of Biological Sciences, Macquarie University, North Ryde, NSW 2109 Australia

Correction to: *Scientific Reports* https://doi.org/10.1038/s41598-020-63622-3, published online 20 April 2020

This Article contains errors in the in-text citations. In the Methods section, under the subheading ‘Climate data and future climate projections’,

“To derive future precipitation and potential evapotranspiration, we used outputs from 16 downscaled global climate models (GCMs) that were part of the Intergovernmental Panel on Climate Change’s (IPCC) Fifth Coupled Model Intercomparison Project, (CMIP5^58^).”

should read:

“To derive future precipitation and potential evapotranspiration, we used outputs from 16 downscaled global climate models (GCMs) that were part of the Intergovernmental Panel on Climate Change’s (IPCC) Fifth Coupled Model Intercomparison Project, (CMIP5^54^).”

And, in the same section,

“Future projected precipitation is an output from the CMIP5 models. Mean annual temperature (Tmean) and annual temperature range (Trange) were the outputs used in conjunction with solar radiation (RA) to model future potential evapotranspiration (PET) using the Hargreaves-Samani equation (Eq. (1)^59,60,61^):

$$ {\text{Equation}}\;(1)\,PET = 0.0023RA\left( {Tmean + 17.8} \right)Trange^{{0.5}}  $$

The Hargreaves-Samani equation was used to calculate the modern AI used in this study^57^ and therefore was also used for the future projections.”

should read:

“Future projected precipitation is an output from the CMIP5 models. Mean annual temperature (Tmean) and annual temperature range (Trange) were the outputs used in conjunction with solar radiation (RA) to model future potential evapotranspiration (PET) using the Hargreaves-Samani equation (Eq. (1)^54,58–61^):

$$ {\text{Equation}}\;(1)\,PET = 0.0023RA\left( {Tmean + 17.8} \right)Trange^{0.5} $$

The Hargreaves-Samani equation was used to calculate the modern AI used in this study^57,58^ and therefore was also used for the future projections.”

