# Supplementary material for: Identifying threshold responses of Australian dryland rivers to future hydroclimatic change
Source: Sci Rep. 2020 Apr 20;10:6653. doi: 10.1038/s41598-020-63622-3 (PMC7171090; doi:10.1038/s41598-020-63622-3)
Supplement: Supplementary file 1 — Supplementary Information. [file 41598_2020_63622_MOESM1_ESM.pdf]

Supplementary Information for

Identifying threshold responses of Australian dryland rivers to future hydroclimatic change

Larkin Z.T.*, Ralph T.J., Tooth S., Fryirs K.A., and Carthey A.J.R.

Zacchary T. Larkin, PhD
Department of Earth and Environmental Sciences, Macquarie University, North Ryde 2109, NSW, Australia
Email: zacchary.larkin@mq.edu.au

This PDF file includes:

Figs. S1 to S3
Table S1

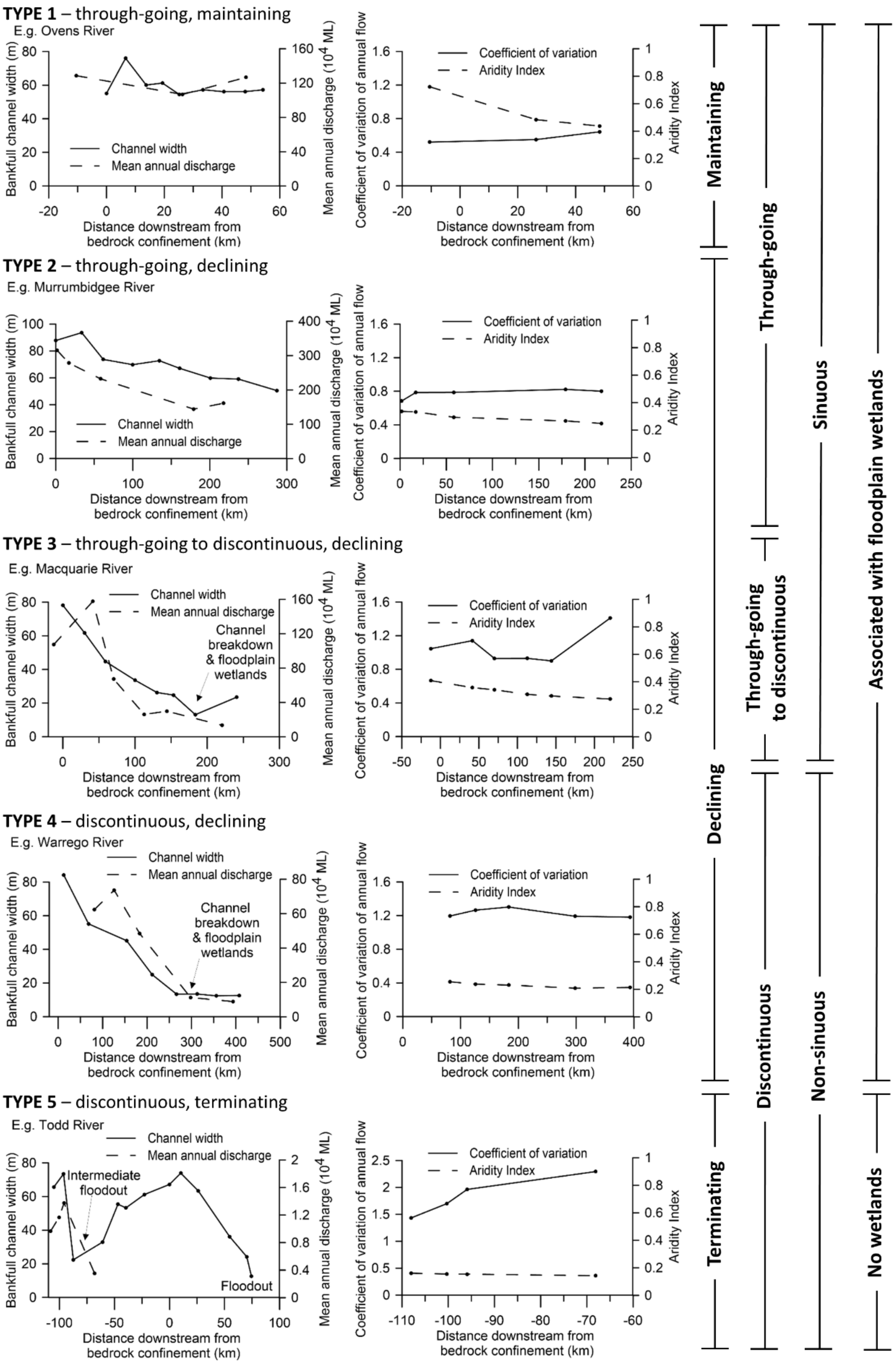

TYPE 1 – through-going, maintaining
E.g. Ovens River
Bankfull channel width (m)
Mean annual discharge (10^4 ML)
Channel width
Mean annual discharge
Distance downstream from bedrock confinement (km)
Coefficient of variation of annual flow
Aridity Index
Coefficient of variation
TYPE 2 – through-going, declining
E.g. Murrumbidgee River
TYPE 3 – through-going to discontinuous, declining
E.g. Macquarie River
Channel breakdown & floodplain wetlands
TYPE 4 – discontinuous, declining
E.g. Warrego River
Channel breakdown & floodplain wetlands
TYPE 5 – discontinuous, terminating
E.g. Todd River
Intermediate floodout
Floodout
Maintaining
Declining
Terminating
Through-going
Through-going to discontinuous
Discontinuous
Sinuous
Non-sinuous
Associated with floodplain wetlands
No wetlands

Supplementary Figure S1. Examples of downstream variation in channel width, mean annual discharge, coefficient of variation of annual flow, and Aridity Index for each of the five Australian dryland river types defined in this study. Maintaining rivers are those that maintain discharge and channel size downstream. Declining rivers are those that decline in discharge and channel size downstream, either gradually (Type 2) or dramatically (Types 3, 4, 5). Through-going rivers (Type 1, 2 and some Type 3) are defined as those that are longitudinally connected and maintain their channel to base level (e.g. another trunk river). Discontinuous rivers (some Type 3, and Type 4 and 5) are those that are characterised by dramatic declines in discharge and channel size downstream of bedrock confinement. They are typically hydrologically discontinuous and characterised by zones of channel breakdown. Some reaches of these systems may be multi-channelled (anabranching/anastomosing or distributary) as a result of decreases in flow and sediment transport capacity. In some Type 3 and Type 4 rivers, flows may reach base level through a reformed channel or overflow, but will have passed through regions of inefficient, unchannelised, or discontinuous flow.

Supplementary Table S1. One-way Analysis of Similarity (ANOSIM) pairwise test statistics. All values significant at $p<0.05$.

River types	R statistic	Significance (P-value)
1, 2	0.781	0.008
1, 3	0.971	0.001
1, 4	0.996	0.005
1, 5	1.000	0.029
2, 3	0.350	0.008
2, 4	0.899	0.002
2, 5	0.956	0.008
3, 4	0.659	0.0004
3, 5	0.787	0.001
4, 5	0.548	0.010

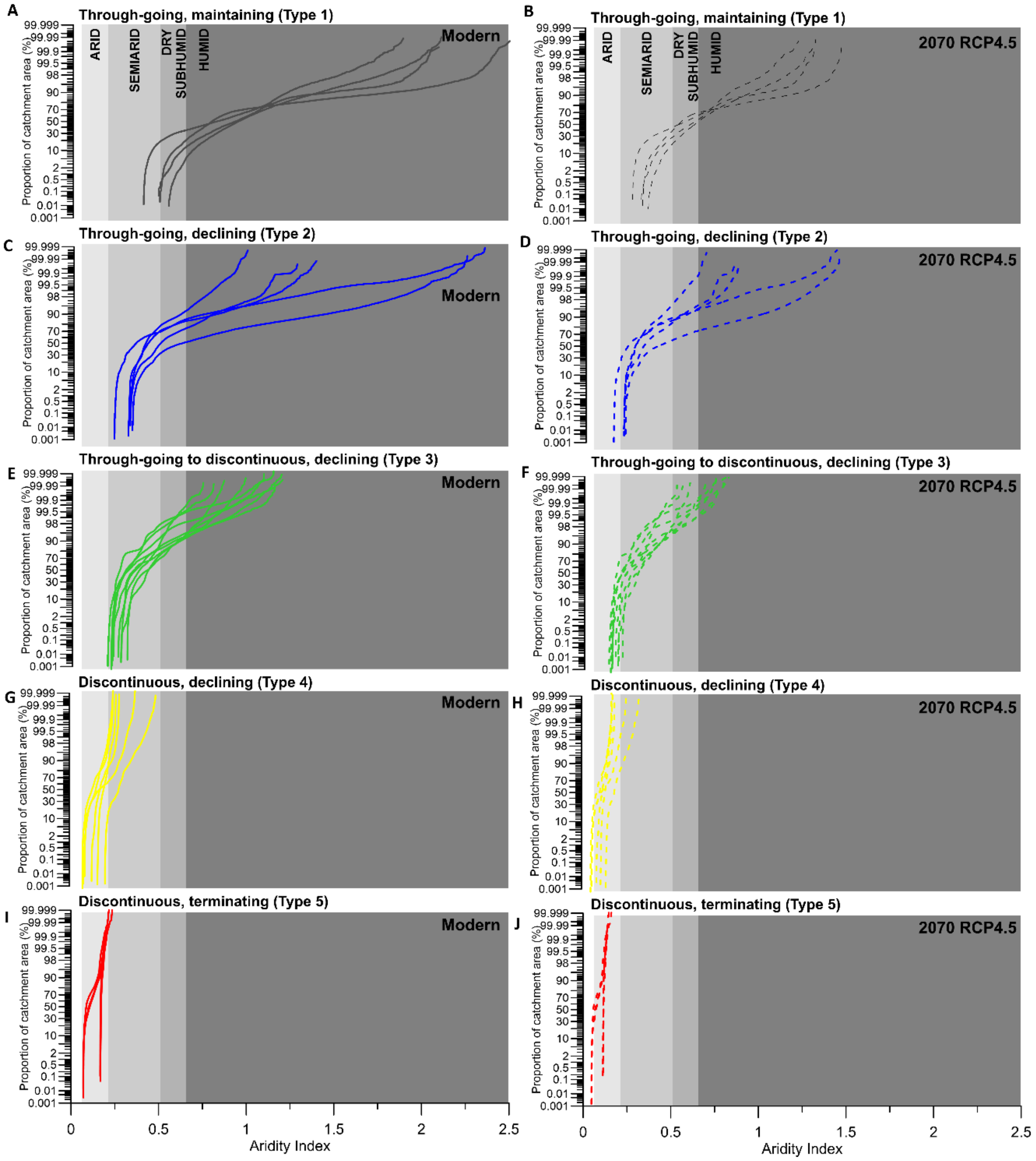

Supplementary Figure S2. The proportions of the catchments of the five river types in different climate zones, as defined by the Aridity Index (AI). (A) Through-going maintaining (Type 1) and (C) through-going, declining rivers (Type 2) presently have higher proportions of their catchment in humid regions, although these proportions will be reduced by 2070 (RCP4.5) (see B and D). (E) Through-going to discontinuous, declining rivers (Type 3) presently have much smaller proportions of their catchments in humid regions, and by 2070 (RCP4.5) may have no humid zones within their catchments (see F). (G) Discontinuous, declining (Type 4) and (I) discontinuous, terminating (Type 5) rivers presently have entirely semiarid or arid catchments, and these catchments will become even more arid by 2070 (RCP4.5) (see H and J). These graphs are plotted with a probability y-axis (upper 10% and lower 10% plotted on log scale) to accentuate the differences between river types for the most humid 10% of each catchment.

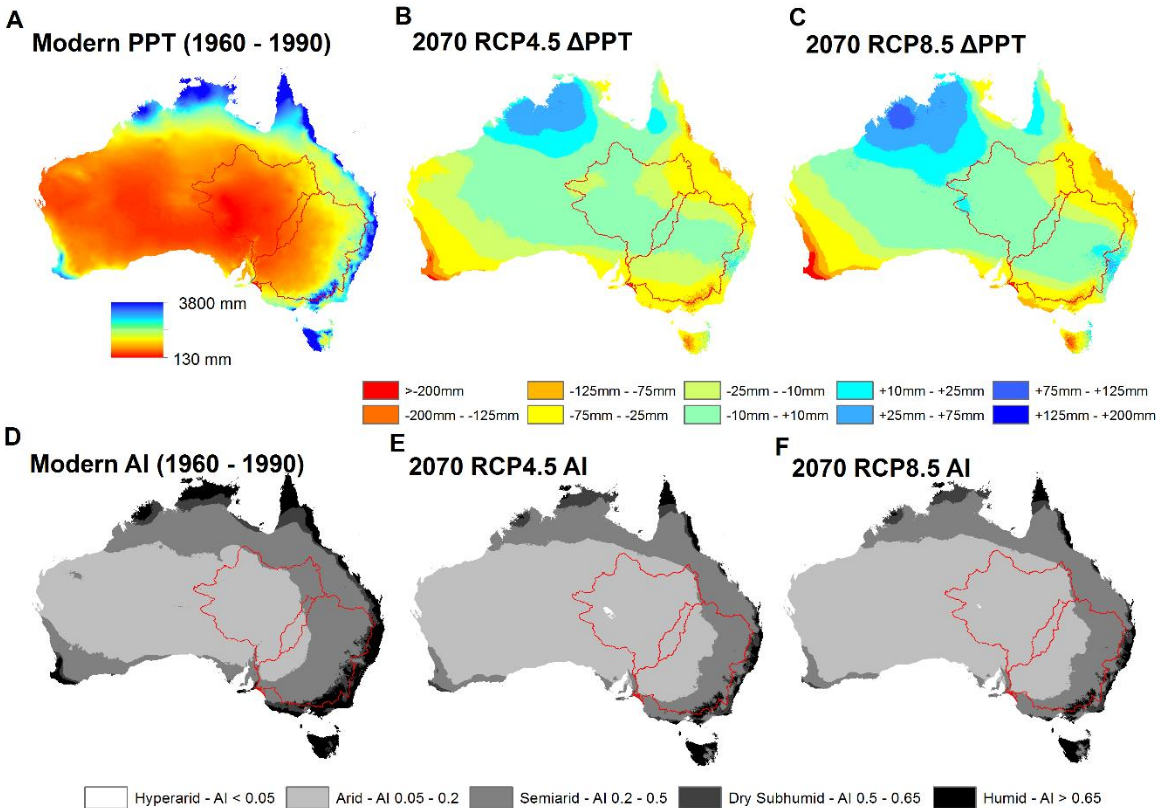

Supplementary Figure S3. (A) Modern distribution of mean annual precipitation (PPT, mm a^{-1}) across the Australian continent. (B) Average projected change in mean annual precipitation (PPT, mm a^{-1}) by 2070 using outputs from 14 IPCC CMIP5 GCMs under RCP 4.5. (C) Average projected change in mean annual precipitation (PPT, mm a^{-1}) by 2070 using outputs from 16 IPCC CMIP5 GCMs under RCP 8.5. (D) Modern Aridity Index (AI) across the Australian continent. (E) Projected (2070 RCP4.5) AI values across the Australian continent. (F) Projected (2070 RCP8.5) AI values across the Australian continent. The Murray-Darling, Bulloo, and Lake Eyre catchment boundaries (the main focus of this study) are marked in red. Map created in ArcMap v.10.2 software (https://desktop.arcgis.com/en/arcmap/).
